# Neural basis of nonanalytical reasoning expertise during clinical evaluation

**DOI:** 10.1002/brb3.309

**Published:** 2015-01-29

**Authors:** Steven J Durning, Michelle E Costanzo, Anthony R Artino, John Graner, Cees van der Vleuten, Thomas J Beckman, Christopher M Wittich, Michael J Roy, Eric S Holmboe, Lambert Schuwirth

**Affiliations:** 1 Department of Medicine, Uniformed Services University of the Health Sciences4301 Jones Bridge Road, Bethesda, Maryland, 20814; 2 National Intrepid Center of Excellence, Walter Reed National Military Medical Center8901 Wisconsin Avenue, Bldg 51, Bethesda, Maryland, 20889; 3 Department of Educational Development and Research, Maastricht University6200 MD, Maastricht, The Netherlands; 4 Mayo Clinic, Division of General Internal Medicine, Department of Internal Medicine, College of MedicineRochester, Minnesota, 55905; 5 American Board of Internal Medicine510 Walnut Street, Suite 1700, Philadelphia, Pennsylvania, 19106-3699, USA; 6 Flinders University, School of MedicineGPO Box 2100, Adelaide, 5001, South Australia, Australia

**Keywords:** Dual-process theory, expertise, functional MRI, medical education, neural efficiency, nonanalytical reasoning

## Abstract

**Introduction:**

Understanding clinical reasoning is essential for patient care and medical education. Dual-processing theory suggests that nonanalytic reasoning is an essential aspect of expertise; however, assessing nonanalytic reasoning is challenging because it is believed to occur on the subconscious level. This assumption makes concurrent verbal protocols less reliable assessment tools.

**Methods:**

Functional magnetic resonance imaging was used to explore the neural basis of nonanalytic reasoning in internal medicine interns (novices) and board-certified staff internists (experts) while completing United States Medical Licensing Examination and American Board of Internal Medicine multiple-choice questions.

**Results:**

The results demonstrated that novices and experts share a common neural network in addition to nonoverlapping neural resources. However, experts manifested greater neural processing efficiency in regions such as the prefrontal cortex during nonanalytical reasoning.

**Conclusions:**

These findings reveal a multinetwork system that supports the dual-process mode of expert clinical reasoning during medical evaluation.

## Introduction

Clinical reasoning entails the cognitive processes that culminate in a diagnosis and treatment plan, and thus is central to almost everything a physician does in practice (Higgs et al. 2008). The “hidden” nature of clinical reasoning renders it difficult to assess through current methods in medical education (Higgs et al. 2008; Schuwirth 2009).Without the ability to directly observe clinical reasoning, a major emphasis of research in clinical reasoning has been the development and testing of theory. Presently, dual-process theory is the leading cognitive theory that has been applied to the construct of clinical reasoning (Norman and Eva 2010). This theory attributes expertise in clinical reasoning to greater use of nonanalytic reasoning, which is believed to be immediate, largely subconscious, and thus difficult or perhaps impossible for subjects to describe (e.g., fast thinking or pattern recognition; Norman and Eva 2010). While medical practitioners regularly use both analytic and nonanalytic reasoning in clinical reasoning tasks, nonanalytic reasoning is believed to correlate most strongly with expertise, yet it is also the more challenging to evaluate (Schmidt and Boshuizen 1993). However, novel neuroimaging techniques may be particularly well-suited to this task.

Cognitive expertise involves chunking of information, or assembling a string of perceptual cues into a more meaningful pattern (de Groot 1965), relying on processes such as working memory (Boreham 1994). Experts are able to generate better problem representation as well as better “next steps or moves” (Simon 1990) in order to select the best diagnostic option (Elstein et al. 1990). Thus, experts differ from novices in how they process information and arrive at an answer, such that experts do not choose more next steps or answers, but the quality of their answers or next steps are superior (Elstein et al. 1990).

In medical education research, think-aloud protocols are a commonly employed means for assessing thought processes while engaging in an activity such as clinical reasoning. Think-aloud method is thought to provide insight into the underpinnings of expertise in physicians (Boreham 1994). However, scholars disagree on the validity of verbally reporting one's thoughts (i.e., think-aloud protocols), because it may interfere with the very act of thinking (Russo et al. 1989; Ericsson 2006). Moreover, think-aloud protocols would be expected to perform better in the assessment of consciously accessible thought processes that are inherent to analytic reasoning (where one actively compares and contrasts options), as opposed to the subconscious processes of nonanalytic reasoning that are believed to be the mainstay of experts engaged in clinical problem solving.

One of the current “gold standards” for assessing the end result of clinical reasoning is multiple-choice questions (MCQs) from professional regulatory authorities such as the American Board of Internal Medicine (ABIM) and National Board of Medical Examiners (NBME). The scores from such high-stakes MCQ tests have evidence of high reliability and validity and allow sampling of a large number of topics during an examination session. MCQs can also isolate tasks such as identifying the most likely diagnosis or the next step in diagnosis or therapy, providing a useful assessment of clinical reasoning, particularly when the questions are vignette-based and require consideration of the optimal diagnosis or treatment. (Schuwirth et al. 2001). However, as MCQs do not allow investigators to observe the thought processes that lead to the final answer, an inability to elucidate the *process* of clinical reasoning can be viewed as a significant limitation.

Given that immediate vocalization of nonanalytic reasoning is difficult, if not impossible, there is a need for other investigative methods to understand this essential aspect of expertise. Functional magnetic resonance imaging (fMRI) is a particularly promising method for enhancing the understanding of nonanalytic reasoning and development of medical expertise, especially when viewed in conjunction with educational theory. fMRI can elucidate otherwise invisible patterns of regional brain activation, acting like a flashlight allows us to see the brain areas and pathways that are inherent to clinical problem solving. Similar assessments in other fields indicate that regions such as the caudate and precuneus appear to play an important role in generating and utilizing perception units or “chunks” when determining the next “best move” in board games, for example, (Wan et al. 2011). There is also some evidence that more skilled or experienced individuals (e.g., experts) demonstrate more efficient neuronal utilization than novices confronted with the same tasks (Neubauer and Fink 2009).

Thus, taking into account both dual-process theory and relevant neuroimaging experience, we compared brain activation patterns for novice and expert physicians in order to discern whether clinical reasoning expertise correlates with distinct activation patterns on functional neuroimaging. We hypothesized that experts and novices would display a shared network of clinical reasoning expertise, as expertise is an adaption built on the foundation developed while one is a novice. Second, extrapolating from other fields, we hypothesized that neural areas such as the precuneus and caudate would demonstrate greater activation in experts as opposed to novices during nonanalytic reasoning. Lastly, the notion of neural efficiency is reportedly a hallmark of skill and expertise (Neubauer and Fink 2009); thus, we hypothesized that experts would display less overall brain activation (more efficient networks activated to accomplish the task) than novices.

## Material and Methods

### Participants

Following completion of written informed consent, board-certified internal medicine attending physicians (experts) and internal medicine interns (novices) with faculty appointments at the Uniformed Services University (USU) participated in the study. Board certification represents the culmination of expertise in medicine and is the culmination of years of medical school and residency education. Hence, we defined board-certified physicians as experts in this study; whereas internal medicine interns, who just completed medical school and were several years away from board certification, were defined as novices. There were several exclusion criteria: presence of shrapnel or surgical metal devices, inability to complete an fMRI due to anxiety or claustrophobia, taking calcium channel blockers (which can impact regional blood flow), or pregnancy. The study protocol and procedures employed were approved by the Institutional Review Boards of the USU and Walter Reed Army Medical Center. The procedures followed were in accordance with the ethical standards of the responsible committee on human experimentation (institutional and national) and with the Helsinki Declaration of 1975, as revised in 2008.

### Demographics

The mean age of the experts was 39.5 ± 7 (range = 32–51 years), including 15 men and two women. For the novices, the mean age was 29.6 ± 2 (range = 28–35 years), including seven men and three women. Experts were significantly older, and had significantly greater years of clinical experience, than novices (*P* < 0.05).

### Measurements

#### Multiple-choice questions

We used validated MCQs from the ABIM and NBME to assess physician performance. These organizations are responsible for certifying or licensing physicians in the United States, and they conduct validity studies to assess the appropriateness of their items by subjecting them to a rigorous internal content review and performance analysis.

We selected cardiology and rheumatology questions for the study, as they represent core domains in internal medicine. The MCQs ask “What is the most likely diagnosis?”, necessitating integration and synthesis of data to answer (i.e., the examination items assessed clinical reasoning). Each participant answered 32 questions: 16 NBME items (United States Medical Licensing Examination [USMLE] Step 2 Clinical Knowledge items) and 16 ABIM items (Maintenance of Certification [MOC]). We selected questions that fit on a single screen and contained only words (i.e., no chest X-rays or other images). In addition, the MCQ format (participants pushed handheld buttons for answer options “A” to “E”) made them ideal for use in the fMRI scanner, eliminating the need for participants to speak, as jaw motion impairs fMRI image interpretation.

#### fMRI Data acquisition

Subjects were scanned on a 3T 750 MRI scanner (General Electric, Milwaukee, WI) with a 32-channel head coil. Acquisitions were performed using an echo-planar imaging (EPI) sequence of 40 contiguous sagittal slices per brain volume (TR = 2000 ms, TE = 25 ms, flip angle = 60° slice thickness = 4.0 mm). In-plane resolution was 3.75 × 3.75 mm (64 × 64 voxels). An fMRI task presentation of the 32 questions was created using E-Prime software (Psychology Software Tools, Inc.) and displayed via a goggle system (Nordic NeuroLab Inc., Milwaukee, WI) while each participant was in the fMRI scanner. The questions were presented in random order for each subject over the course of four fMRI acquisition runs, with eight questions per run. The mean run length (± standard deviation) was 392 ± 62 sec. During the same imaging session, a high-resolution T1-weighted image was acquired for anatomical reference (three dimensional GRE; TR = 6.6 ms, TE = 2.5 ms, flip angle = 12°). This image consisted of 312 sagittal slices with a slice thickness of 0.6 mm and an in-plane resolution of 0.468 × 0.468 mm (512 × 512 voxels). For voxel-wise analysis on whole brain data, we controlled false positive rates per map at alpha=0.05, using random-effects models and consistent with prior work.

### Procedure

Before entering the fMRI scanner, participants were formally trained in procedures for answering MCQs in the scanner. Each MCQ was projected in three phases. In the first phase, the stem (question) appeared (“reading” phase), ending with “what is the most likely diagnosis?” or a related diagnostic question, but not displaying answer options “A” to “E”. Each participant was given a maximum of 60 sec to read the stem, or could push any button to move on to the answer options (the second or “answering” phase) more quickly. Participants were then given 7 sec to choose an answer option using the finger response items. The final “reflection” phase ensued, in which participants were instructed to silently reflect on how they arrived at the diagnosis utilizing analytical reasoning processes (“how did you establish the diagnosis for this item?”), which they did for 14 sec, before the next question was presented. The reflection phase thus was characterized by analytic thinking about how they chose the answer they did (e.g., actively comparing and contrasting alternatives). Before entering the scanner, participants received training on how to analyze their thinking (a think-aloud procedure).

### fMRI Data analysis

All fMRI data were processed using the AFNI software package in accordance with previously published methods (Cox 1996; Durning et al. 2012). The participant's EPI scans were preprocessed by first removing the three volumes (6 sec) from each 4D time series. Next the scans were corrected for slice timing and motion then coregistered to the T1 anatomical image (anatomic scans were registered to Talairach space). The images were spatially smoothed using 8-mm full-width at half-maximum Gaussian kernel and converted to percent-change-from-mean. For the first level analysis, the four datasets for each subject were concatenated. The “answer” times varied from question to question (depending on how quickly the participant answered) and were modeled with a gamma-variate function with variable duration and variable relative amplitude (amplitude variation was based on duration variation). The “reflection” time was constant at 14 sec and was modeled with a nonvariable gamma-variate. The GLM analysis determined the significance of these model time courses, along with head motion parameters, to generate *β* coefficients and *t* statistics for each voxel, for the contrast of interest: answer phase relative to reflection (answer > reflection) which isolates nonanalytical reasoning: answering (utilizing both analytical and nonanalytical reasoning)—reflection (analytical reasoning)(Chen et al. 2012). Second-level analysis across all subjects was then performed using linear mixed-effects modeling conducted on the individual contrast for experts and novices separately. These comparisons were used in the conjunction analysis (Price and Friston 1997) to examine brain regions with similar levels of activation, versus those with significantly different levels of activation, between the two groups for answer > reflection. Results of the second-level analyses were corrected for multiple comparisons using family wise error (FWE) correction (from a Monte Carlo simulations using AFNI's 3dClustSim) to achieve corrected *P* values (*P* < 0.05) based on cluster size.

## Results

### fMRI Conjunction analysis

**Figure 1:**
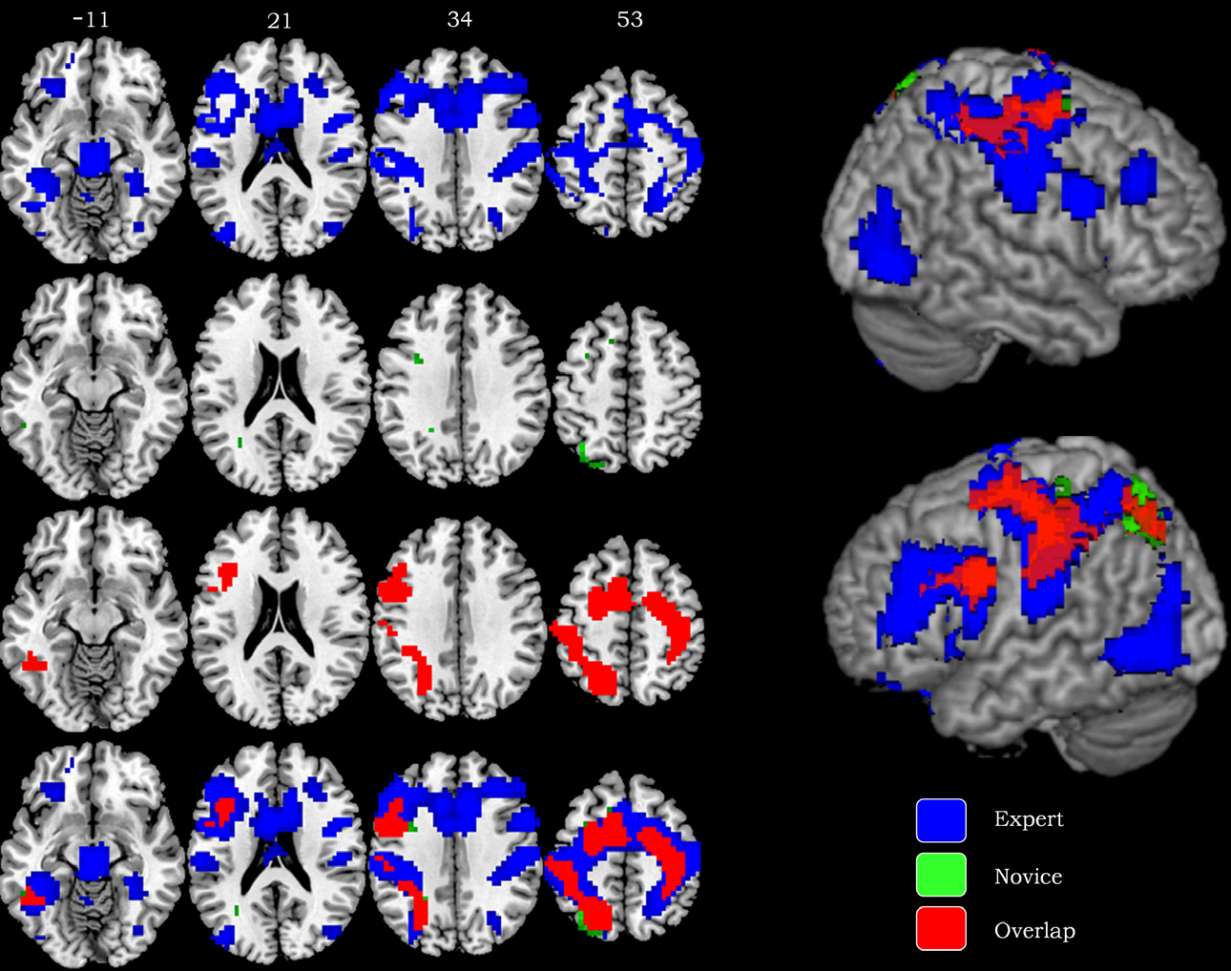
Whole brain analysis of experts and novices during nonanalytic clinical reasoning (answering > reflection). Axial slices with corresponding Talairach coordinates indicate unique and shared activation patterns for experts and novices. The results demonstrate nonoverlapping activations for experts in blue and nonoverlapping activation for novices in green. The activations shared by both groups are represented in red. All results are thresholded at FWE corrected *P* < 0.05.

Whole brain analysis revealed a common network, with similar levels of activation, between the two groups involving the bilateral precentral gyrus, bilateral middle frontal gyrus, bilateral dorsomedial prefrontal cortex (DMPFC), left dorsolateral prefrontal cortex (DLPFC), bilateral postcentral gyrus, bilateral inferior parietal lobule, left superior parietal lobule, left precuneous, left middle temporal gyrus, and left fusiform gyrus (Table 1, Fig.1). Areas of significantly greater activation in experts were the left ventrolateral prefrontal cortex (VLPFC), left lateral orbitofrontal cortex (OFC), right superior parietal lobule, right inferior occipital gyrus, bilateral middle occipital gyrus, bilateral insula, bilateral lentiform nucleus, bilateral dorsal anterior cingulate cortex (ACC), bilateral cerebellum, bilateral thalamus, and bilateral parahippocampal gyrus. The sole area in which novices demonstrated significantly greater activation than experts was the ventral anterior cingulate cortex (Table 1, Fig.1).

**Table 1:** Peak activations during nonanalytic reasoning

Region		Experts					Novices				
Ans>Refl	Brod. no.	Hemi	*x*	*y*	*z*	*t* score	Hemi	*x*	*y*	*z*	*t* score
Precentral Gyrus*	BA4	L	−36	−17	60	5.8	L	−37	−23	59	3.0
		R	40	−19	54	8.1	R	37	−20	54	4.8
Middle Frontal Gyrus*	BA6	L	−21	−3	56	7.0	L	−28	−7	57	5.1
		R	30	−9	58	6.7	R	26	−9	56	4.5
Medial Frontal Gyrus (DMPFC)*	BA8	L	−2	16	48	6.7	L	−11	4	48	4.8
		R	1	5	51	5.9	R	11	15	43	3.0
DLPFC*	BA9	L	−47	28	29	9.8	L	−49	6	37	3.9
DLPFC*	BA46	L	−43	30	23	8.2	L	−40	20	23	3.2
IFG(VLPFC)	BA45	L	−52	7	23	6.2					
		R	54	10	24	5.4					
OFC (lateral)	BA47	L	−31	28	−4	7.1					
Postcentral Gyrus*	BA3	L	−40	−22	55	6.3	L	−41	−27	56	2.9
		R	47	−23	48	9.1	R	39	−27	54	4.3
Inferior Parietal Lobule*	BA40	L	−36	−39	48	6.7	L	−40	−40	44	4.0
		R	32	−42	50	6.8	R	33	−42	50	4.0
Superior Parietal Lobule*	BA7	L	−28	−53	58	5.5	L	−28	−63	55	5.5
		R	27	−55	51	4.2					
Precuneus*		L	−28	−68	37	5.9	L	−23	−60	48	4.9
Middle Temporal Gyrus*	BA39	L	−29	−65	30	5.1	L	−28	−58	28	3.4
Fusiform*	BA37	L	−40	−56	−8	6.3	L	−46	−50	−10	3.2
		R	29	−41	−15	4.1					
Inferior Occipital Gyrus	BA19	R	43	−77	−4	4.8					
Middle Occipital Gyrus	BA39	L	−44	−76	14	4.0					
		R	32	−82	14	3.7					
Insula	BA13	L	−29	16	14	5.3					
		R	29	25	10	5.9					
Lentiform Nucleus		L	−17	5	3	5.7					
		R	11	4	0	5.7					
Dorsal ACC	BA32	L	−3	20	40	5.6					
		R	5	19	40	5.3					
Ventral ACC	BA24						L	−22	6	43	3.6
Cerebellum		L	−17	−54	−28	5.8					
		R	26	−61	−30	4.9					
Thalamus		L	−7	−17	−7	4.8					
		R	6	−17	−8	4.9					
Parahippocampal Gyrus		L	−34	−36	−10	3.4					
		R	33	−38	−9	3.9					

Nonanalytic reasoning (Answering > Reflection) in experts (left panel) and novices (right panel). Asterisk (^*^) denotes regions of similar levels of activation as revealed by conjunction analysis. Brodmann's areas and laterality (hemisphere) are provided in addition to coordinates given in Talairach space. All results are based on FWE correction *P* < 0.05 and *t* scores are indicated.

### fMRI Direct group comparisons

**Figure 2:**
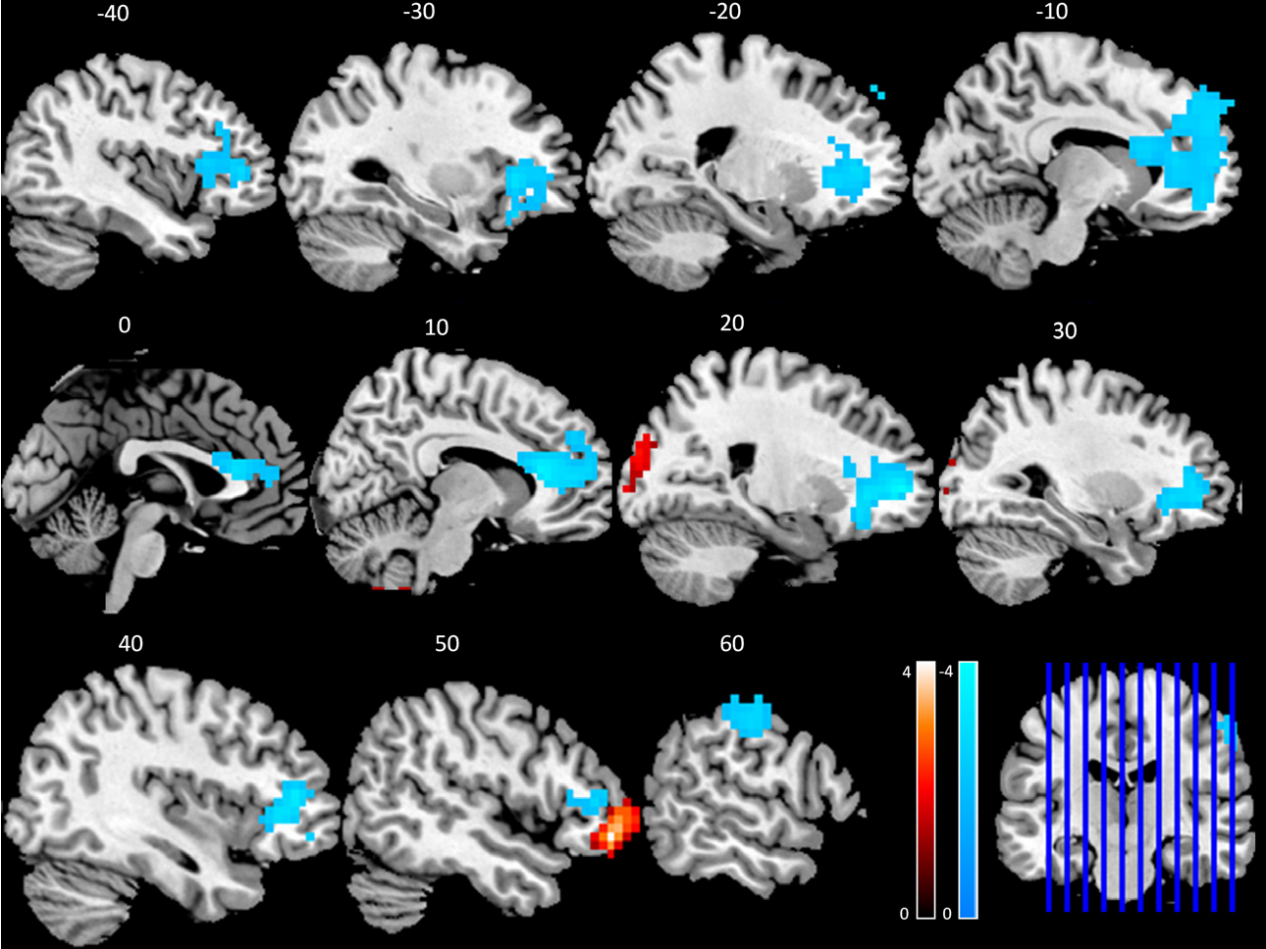
Direct comparisons between experts and novices (Expert > Novice) during nonanalytic clinical reasoning. Warm colors denote regions where experts demonstrate greater responses and cooler colors reveal regions where novices demonstrate greater responses during nonanalytic clinical reasoning (answering > reflection). Sagittal slices are presented with corresponding Talairach coordinates and all results are thresholded at FWE corrected *P* < 0.05. The color bars indicate *t* scores and the coronal slice presents orientation of sagittal results.

When directly comparing experts and novices for the magnitude of differences between the two groups, experts demonstrated significantly less activation relative to novices in the right postcentral gyrus, bilateral DLPFC, DMPFC, bilateral ventromedial prefrontal cortex, bilateral lateral OFC, bilateral medial OFC, ventral ACC, and dorsal ACC. Significantly greater activation in experts compared to novices was evident in the rostrolateral prefrontal cortex and cuneus. (Table 2, Fig.2).

**Table 2:** Direct comparison between experts and novices (answer > reflection)

		Expert					Novice				
Region	Brod. no.	Hemi	*x*	*y*	*z*	*t* score	Hemi	*x*	*y*	*z*	*t* score
Ans > Refl											
Postcentral Gyrus	BA1						R	56	−16	48	−2.95
DLPFC	BA9	L	−48	28	28	−3.030					
DLPFC	BA46	L	−40	36	10	−2.66	R	41	35	10	−3.699
DMPFC	BA9	L	−8	50	36	−4.295	R	15	44	27	−3.037
vmPFC	BA10	L	−18	47	7	−3.099	R	19	50	1	−3.717
Ventral ACC	BA24	L	−8	17	22	−3.500	R	4	29	13	−3.77
lOFC	BA47	L	−28	30	−4	−3.110	R	26	30	−2	−3.05
mOFC	BA11	L	−16	46	−10	−2.460	R	24	27	−12	−3.179
Dorsal ACC	BA32	L	−8	22	20	−2.96	R	6	28	22	−2.76
RLPFC	BA10						R	46	43	−6	3.55
Cuneus	BA18						R	17	−84	25	2.82

Negative *t* scores are indicative of areas demonstrating significantly lesser activation for experts than for novices, while positive *t* scores reveal areas of greater activation in experts compared to novices. Brodmann's areas and laterality (hemisphere) are provided in addition to coordinates given in Talairach space. All results are based on FWE correction *P* < 0.05.

## Discussion

To our knowledge, this is the first study to utilize functional neuroimaging to study nonanalytic reasoning during evaluation in the field of medicine. We explored the functional neuroimaging of expert and novice performance using the current gold standard for competency assessment, validated, vignette-based MCQs. We report that novices and experts share a common neural network, but also show some significant differences in regional brain activation, during nonanalytical reasoning (Fig.1). Experts demonstrate neural-processing efficiency in regions such as the prefrontal cortex (Fig.2), which may buttress dual-process theory, and help to elucidate neural networks that represent expertise. This may ultimately enable the identification of fMRI biomarkers of effective clinical reasoning, which could facilitate educational interventions to improve desired regional brain activation in order to reduce cognitive errors.

### Shared network

The results support our hypothesis that experts and novices share a common network of activation. Experts and novices demonstrate similar levels of activation in the motor (BA4) and premotor (BA6) regions postulated to be critical to clinical reasoning (Fletcher and Carruthers 2012). The DMPFC and left lateral DLPFC both also showed similar activation levels for both groups. The former is reportedly involved in self-referential evaluation (Northoff and Bermpohl 2004), which may be critical in understanding and making inferences (Amodio and Frith 2006), in turn contributing to nonanalytical information processing. The latter is thought to be involved in attention shifting and control, selection (Sylvester et al. 2003) modulation of self-control (Figner et al. 2010), and cognitive flexibility (Braver et al. 2009).

We also found some posterior brain regions that were active in both groups. The inferior parietal lobule is involved in the validation of deductive reasoning, and the fusiform may mediate the integration of information with a working premise (Fangmeier et al. 2006). The postcentral gyrus has been shown to be connected with mental preparation for successful problem solving (Tian et al. 2011). We expected the precuneus to show greater activation in experts, but identified similar activations in both groups, suggesting that even novices were employing some pattern recognition, or nonanalytic reasoning in answering. This is not entirely surprising, as our “novices” have completed both college and medical school, and are currently engaged in postgraduate medical education, so that they are not entirely new to the field. This may represent an intermediate step in the development of expertise, as experts also demonstrate distinctive features during nonanalytical reasoning (see next section).

### Expert patterns

Among the regions in which experts evince greater activation is the VLPFC, which mediates working memory retrieval (Wolf et al. 2006). The left VLPFC has been implicated in cognitive control of memory including task switching, knowledge-based retrieval, integration of past events, and resolution of task interference (Badre and Wagner 2006), whereas the right facilitates the update of action plans which may be part of the automaticity of expertise. (Levy and Wagner 2011). Experts also differentially activate the lateral OFC, which may prepare for outcome changes (Windmann et al. 2006) that contribute to decision making (Kringelbach 2005). This region may thus facilitate connections between prestored knowledge and the new clinical scenario.

Experts demonstrate greater activation in several regions that may orchestrate the “chunking” or pattern recognition believed to be integral to nonanalytic reasoning, including the inferior occipital gyrus, middle occipital gyrus (Ruff et al. 2003) and parahippocampal gyrus (Aguirre et al. 1996). On the other hand, we believe that differential expert activation of the insula, a region involved in the integration of sensory information (Medford and Critchley 2010) as well as empathy, emotion, and the processing of uncertainty (Singer et al. 2009), may be a manifestation of the “gut instinct” that comes from experience. Our experts also showed greater recruitment of the dorsal anterior cingulate cortex, a region reportedly involved in conflict resolution during error detection (Braver et al. 2001). The ACC processes cognitive and affective representations, in addition to sensory and motor information, in order to evaluate error (Bush et al. 2000). Activation of ACC in tandem with the insula supports a recent model of multimodal integration in response selection (Medford and Critchley 2010). Cultivation of this functional network may therefore spawn better-informed choices and minimize diagnostic error.

Another region of unique activation in experts is the bilateral cerebellum. Recent studies have suggested that the cerebellum may process not only motor control but also mediate cognitive control in the form of rule retrieval (Crescentini et al. 2011; Balsters et al. 2012), which may contribute to the automaticity of nonanalytic clinical reasoning. Although we did not find activation in the caudate as we had predicted with experts, the lentiform nucleus of the basal ganglia was more active. Lesion studies suggest this region is involved in drive and initiative (Brown et al. 1997). Lastly thalamic activation suggests an elevated intensity, alertness, and arousal (Sturm et al., 1999) unique to experts.

### Novice patterns

The ventral ACC was the only region where novices showed greater activation than experts. The ventral ACC is involved in the emotional response to error (Braver et al. 2001), so this may represent an emotional response (Etkin et al. 2011).to their greater uncertainty when challenged with MCQs.

### Neural efficiency and expertise

Direct comparisons between the expert and novice group revealed significantly less activation in frontal and selective posterior regions for the experts. The relative reduction of activity in these areas may mean that experts are more efficiently able to incorporate these areas into their diagnostic decisions, whereas novices require more cognitive effort to accomplish this. Thus, our findings suggest that experts may make better “first moves” because they can more efficiently activate relevant areas of the brain for the task of clinical reasoning.

Our results reveal relative deactivation of the DLPFC of the experts, supporting our hypothesis. As previously discussed, the DLPFC is essential to attention shifting, working memory and inhibitory control (e.g., Glascher et al. 2012) and such neural efficiency suggests that experts may require fewer neural resources to accomplish the task demands. In addition, the relative reduced activation in the DMPFC suggests experts are more efficient with referential processing (Yaoi et al. 2009), and the evaluation of the self's qualities within the goal of the moment (Beer et al. 2010). The relative LOFC deactivation in experts suggests neural efficiency when weighing outcome uncertainty and probabilistic choices (Windmann et al. 2006). In addition, the efficiency in the dorsal anterior cingulate of the experts suggests that error evaluation requires less effort to accomplish a high level of performance (Braver et al. 2001). The relative reduction in the lOFC and ACC suggests that although these regions are uniquely recruited by experts during nonanalytical reasoning (see Table 1), they are more efficient in processing compared to novices.

Significant differences in several frontal regions were only revealed during direct comparisons between the groups. The experts demonstrated significantly less activation in the ventromedial prefrontal cortex (VMPFC), a region in processing of metacognitive representations such as outcome selection (Amodio and Frith 2006). Our results also revealed a relative deactivation in the ventral ACC of experts, suggesting that they require less neural resources when affectively evaluating possible errors (Etkin et al. 2011). Neural efficiency is also present in the medial OFC (Windmann et al. 2006) of the experts. Notably, the medial OFC is also reportedly involved in empathy and compassion (Klimecki et al. 2012), consistent with the efficiency demonstrated in the postcentral gyrus, a region that mediates advanced mentalizing about emotion and its relationship to empathy, which lead to a greater ability to empathize (Hooker et al. 2008). Collectively, these regions are sensitive to the development of social cognition and perhaps serve as a locus for professionalism. This could, in other words, indicate that experts are activating examples of actual patients with answering MCQ vignettes and thus professionalism issues are being considered and/or incorporated into their answers.

Not only do our results support the notion that expertise is mediated by neural efficiency in terms of deactivation, but we demonstrated that such refinement also requires selective heightened activation relative to novices. Although we had predicted that the precuneus and caudate would demonstrate greater activation in experts compared novices, our results revealed instead the rostrolateral prefrontal cortex (RLPFC) and the cuneus as regions significantly greater in the experts. The RLPFC is a region involved in cognitive processing of abstract, stimulus-independent information, in addition to planning and prospective memory (Gilbert et al. 2006; Wagner et al. 2006; Rubens and Zanto 2011). The right lateralization is related to processing demands (Bunge et al. 2009) and acts in concert with the hippocampus during relational encoding (Wendelken and Bunge 2010). The cuneus has been associated with reasoning (Ruff et al. 2003), specifically deductive reasoning (Barbey and Barsalou 2010) which may utilize visuospatial information.

Limitations of this investigation include our relatively small sample and the lack of a period of formal rest or inactivity; however, as we sought to capture the construct of reasoning, and in particular the construct of nonanalytic reasoning, we believe that comparing answering and reflecting phases would result in more meaningful, task-specific findings.

## Conclusions

Implications of our work include the idea that there may be a functional neuroimaging pattern or “locus” of clinical reasoning expertise during educational evaluation. We believe that the activation of multiple areas of the brain is likely due to the complexity of the task (clinical reasoning). Thus, we may also have identified a multiregion expertise network for clinical reasoning as both novices and experts activated the same areas during educational evaluation, with rare exception. Due to the complexity of clinical reasoning, it may be that such a network is needed for seemingly effortless (or at least more efficient) processing of complex data from patients to arrive at a diagnosis. In addition experts had less activation in several areas of the frontal lobe when answering MCQs supporting the notion of neural efficiency.

The differences and similarities between experts and novices suggest that there is a core network of regions that play a role in moving from novice to expert in clinical reasoning. Indeed our results support a recent review in which expertise was characterized within a two-stage framework with decreased activity and cerebral functional reorganization relating to chunks and knowledge structure (Guida et al. 2012). This is encouraging as it suggests that, if reproducible, future work may be able to plot the trajectory of expert performance and provide more specific feedback to individuals based on the pattern of functional neuroactivation. Such development of single subject analysis was recently discussed in the context of clinical diagnosis (Bullmore 2012), and although such approaches are not yet available, it has potential to contribute to the mitigation of diagnostic errors. In summary, our study utilized established educational theory, two separate participant groups (experts and novices), as well as task items (MCQs) that have been well-validated for assessing clinical reasoning, to provide evidence that expertise involves a distributed and refined brain network during nonanalytical reasoning.
